# Impact of appliance design and the magnitude of maxillary expansion on facial soft tissue: systematic review

**DOI:** 10.1590/1807-3107bor-2026.vol40.085

**Published:** 2026-11-09

**Authors:** Livia Maria Alves Valentim da SILVA, Jéssica Marcela de Luna GOMES, Luy de ABREU-COSTA, João Pedro Justino de Oliveira LIMÍRIO, Victor Perinazzo SACHI, Wirley Gonçalves ASSUNÇÃO, André Pinheiro de Magalhães BERTOZ, Eduardo Piza PELLIZZER

**Affiliations:** (a) Universidade Estadual Paulista – Unesp, School of Dentistry, Department of Pediatric and Social Dentistry, Araçatuba, SP, Brazil.; (b) Universidade Estadual Paulista – Unesp, School of Dentistry, Department of Dental Materials and Prosthodontics, Araçatuba, SP, Brazil.; (c) Universidade Estadual Paulista – Unesp, School of Dentistry Department of Diagnosis and Surgery, Araçatuba, SP, Brazil.

**Keywords:** Palatal Expansion Technique, Child, Face, Orthodontics

## Abstract

Rapid maxillary expansion (RME), which produces skeletal widening of the palate, can affect the overlying soft tissues. This systematic review aimed to evaluate the impact of appliance design and the magnitude of RME after 6 months on facial soft tissue changes in children with maxillary constriction (CRD420251053665). Searches were conducted using the PubMed/MEDLINE, Cochrane Library, Scopus, Embase, Web of Science and ClinicalTrials.gov databases until April 2026. Randomized and non-randomized clinical trials (RCTs and NRCTs, respectively) assessing facial soft tissue changes in children undergoing RME were included. Studies involving surgical expansion, mini-implants, or patients with craniofacial syndromes or systemic growth-related conditions were excluded. Linear and angular facial measurements before and after treatment were synthesized according to the type of appliance and magnitude of expansion. Quality of evidence was assessed using the Risk of Bias 2 and Cochrane Risk Of Bias In Non-randomised Studies – of Interventions tools, and the certainty of the evidence was determined using the GRADE system. The search identified 796 studies, of which 18 studies met the inclusion criteria; six were judged to have a low risk of bias. The GRADE assessment rated the certainty of the evidence as low and very low for RCTs and NRCTs, respectively. The studies demonstrated small linear increases in the nasal and perioral regions (1–2.5 mm) and mild angular changes (3–4°), with greater changes observed with banded expanders and greater expansion magnitudes. Differences between the types of expanders were minimal when the final expansion was similar. Although expansion magnitude, rather than appliance design, was the main determinant of soft tissue changes, the clinical and esthetic relevance of these changes remains uncertain.

## Introduction

Rapid maxillary expansion (RME) is a well-established and effective technique for correcting transverse maxillary deficiencies, as it promotes separation of the midpalatal suture.^1^ Beyond skeletal correction, several studies have reported potential functional benefits of RME, such as improvements in nasal breathing and masticatory function,^2^ as well as changes in the facial soft tissues.^3^

Facial soft tissue changes following RME have been consistently reported in children. Although the most frequently described alterations include increased upper lip length and widening of the nasal base, these changes have been documented through direct measurements, two-dimensional (2D) cephalometric analyses, and three-dimensional (3D) imaging techniques,^4^ substantial methodological heterogeneity exists in the literature, precluding direct comparison and interpretation of the available results.

The most comprehensive systematic review on this topic, published in 2018,^5^ reported that the majority of linear and volumetric soft tissue changes following RME exhibited mean magnitudes of less than 1 mm, a magnitude below the threshold of clinical detection and esthetic perceptibility, suggesting a limited overall impact. Nevertheless, despite including a meta-analysis, this review was unable to robustly assess the influence of specific variables, such as appliance design or the effective magnitude of expansion achieved, factors that may influence the effects of RME.

More recent evidence involving similar treatment protocols with skeletal anchorage further highlights this variability. While some studies reported slight thinning and posterior repositioning of the upper lip, increased projection in the cheek region, and minor changes in nasal dimensions, other studies did not report clinically perceptible alterations in the facial profile.^6^

Thus, determining the extent to which the observed soft tissue changes are truly attributable to the therapeutic protocol is crucial given the continuous skeletal and dentoalveolar growth of children undergoing treatment.^7^ Accordingly, part of the changes identified may merely reflect expected physiological development. Nevertheless, evidence indicates that certain osseous structures, such as the height and length of the piriform aperture, remain altered following RME, suggesting that the intervention exerts a direct effect independent of natural growth processes.^8^

In addition to updating the current evidence, this systematic review seeks to clarify whether facial soft tissue changes following RME are primarily influenced by the design of the expansion appliance or by the magnitude of expansion achieved. The findings suggest that banded expanders may produce slightly greater changes in the nasal and perioral regions; however, these differences appear to be mainly related to the greater magnitude of expansion rather than the appliance design itself. When similar expansion magnitudes are achieved, these differences tend to diminish, indicating that the amount of expansion may be the main determinant of facial soft tissue changes. Therefore, this systematic review aimed to evaluate the impact of appliance design and the magnitude of RME on facial soft tissue changes in children with maxillary constriction.

## Methods

### Protocol and registration

This systematic review was conducted in accordance with the Preferred Reporting Items for Systematic Reviews and Meta-Analyses (PRISMA) guidelines^9^ and was registered in the PROSPERO database (CRD420251053665).

### Inclusion and exclusion criteria

A population, intervention, comparison, outcomes, and study design (PICOS) question was formulated: “Do appliance design and the magnitude of RME influence facial soft tissues in children with maxillary constriction?” The population (P) comprised children diagnosed with maxillary constriction according to the diagnostic criteria reported in the included studies, including clinical or radiographic assessment, posterior crossbite, or the clinical need for RME. The included participants had mean ages ranging from approximately 8 to 16 years, with an overall mean age of approximately 11.3 ± 2.2 years. The intervention (I) was RME. The comparison (C) was the pre-expansion period. The outcomes (O) of interest were linear and angular measurements of facial soft tissues. The study designs (S) included randomized and non-randomized clinical trials.

#### Exclusion criteria

Studies were excluded if they involved the use of orthodontic mini-implants, employed surgically assisted or orthopedic expansion approaches, or included patients with craniofacial syndromes or systemic conditions that could affect craniofacial growth.

## Information sources and search strategy

An electronic search was performed in the PubMed/MEDLINE, Cochrane Library, Scopus, Embase, and ClinicalTrials.gov databases by two independent reviewers (L.M.A.V.S. and L.A.C.) to identify articles published until April 2026, with no language or time restriction. For each electronic database, a specific search strategy was developed, as described in Table 1.

**Table 1:** Search terms used across the databases.

Databases	Set of terms	Filter
PubMed/MEDLINE	(“palatal expansion technique”[MeSH Terms] OR (“palatal”[All Fields] AND “expansion”[All Fields] AND “technique”[All Fields]) OR “palatal expansion technique”[All Fields] OR (“maxillary”[All Fields] AND “expansion”[All Fields]) OR “maxillary expansion”[All Fields] OR ((“rapid”[All Fields] OR “rapidities”[All Fields] OR “rapidity”[All Fields] OR “rapidness”[All Fields]) AND (“palatal expansion technique”[MeSH Terms] OR (“palatal”[All Fields] AND “expansion”[All Fields] AND “technique”[All Fields]) OR “palatal expansion technique”[All Fields] OR (“maxillary”[All Fields] AND “expansion”[All Fields]) OR “maxillary expansion”[All Fields]))) AND ((“face”[MeSH Terms] OR “face”[All Fields] OR “facial”[All Fields] OR “facials”[All Fields]) AND “soft”[All Fields] AND (“tissue s”[All Fields] OR “tissues”[MeSH Terms] OR “tissues”[All Fields] OR “tissue”[All Fields]))	No filters applied
Cochrane Library	((maxillary expansion OR rapid maxillary expansion) AND (facial soft tissues)) in Title Abstract Keyword - (Word variations have been searched)	No filters applied
Scopus	Information of advanced query TITLE-ABS-KEY ( maxillary AND expansion OR rapid AND maxillary AND expansion AND facial AND soft AND tissues )	No filters applied
Embase	SourcesEmbase, MEDLINE, Preprints, Clinical Trials	No filters applied
	Query(‘maxillary expansion’/exp OR ‘maxillary expansion’ OR ((‘maxillary’/exp OR ‘maxillary’ OR ‘maxillary’/exp OR maxillary) AND expansion) OR ‘rapid maxillary expansion’/exp OR ‘rapid maxillary expansion’ OR ((‘rapid’/exp OR ‘rapid’ OR ‘rapid’/exp OR rapid) AND (‘maxillary’/exp OR ‘maxillary’ OR ‘maxillary’/exp OR maxillary) AND expansion)) AND (‘facial soft tissues’ OR (facial AND soft AND (‘tissues’/exp OR ‘tissues’ OR ‘tissues’/exp OR tissues)))	
	Mapped terms’tissues’ mapped to ‘tissues’, term is exploded	
Web of Science	((maxillary expansion OR rapid maxillary expansion) AND (facial soft tissues))	
Clinical Trials	maxillary expansion | Other terms: rapid maxillary expansion	No filters applied

## Study selection and data extraction

Following the systematic literature search, all identified citations were imported into the Rayyan QCRI (https://rayyan.ai/), and duplicates were removed. Titles and abstracts were independently screened by two reviewers (L.M.A.V.S. and L.A.C.), who screened the studies for elgibility and applied the predefined eligibility criteria. In cases of disagreement between the two reviewers, a third reviewer (J.M.L.G.) was consulted, and consensus was reached through discussion.

The full texts of potentially eligible studies were retrieved and independently reviewed in detail by two reviewers. One author (L.M.A.V.S.) was responsible for extracting the qualitative or quantitative data from the included studies, and two authors (L.A.C. and J.M.L.G.) reviewed all collected information. In addition to the outcomes, the following data were extracted from each study: author and year of publication, study design, number of patients, follow-up period, mean age and sex distribution, method used to diagnose maxillary constriction, appliance design, magnitude of expansion, facial analysis method, results, and intervention effect.

In the case of missing or unclear information, assumptions were made based on descriptions provided in the methodology section or the study authors were contacted for clarification. The information was recorded as “not reported” upon receiving no response. The results were grouped into linear and angular facial soft tissue measurements according to the type of appliance and magnitude of maxillary expansion.

## Risk of bias assessment

Two reviewers (L.M.A.V.S. and L.A.C.) independently assessed the methodological quality and risk of bias of randomized clinical trials using the Cochrane Risk of Bias 2 (RoB 2), which evaluates five domains. Each domain was judged as low risk, high risk, or some concerns, resulting in an overall risk-of-bias judgment for each study. For non-randomized intervention studies, the Cochrane Risk Of Bias In Non-randomised Studies – of Interventions (ROBINS-I) was applied, assessing the risk of bias across the pre-intervention, intervention, and post-intervention phases based on seven specific domains. The certainty of the evidence regarding facial soft tissue changes was assessed using the GRADE approach.

## Additional Analysis

An additional analysis was performed to assess intra-rater agreement during the individual searches conducted in the study selection process, using the kappa coefficient.

## Results

### Study selection

A total of 796 records were identified across multiple databases, including PubMed (n = 185), Cochrane Library (n = 22), Scopus (n = 133), Embase (n = 120), Web of Science (n = 231), and ClinicalTrials (n = 105). After eliminating 297 duplicates, 499 records underwent title and abstract screening (phase 1), of which 449 were excluded. Subsequently, 50 full-text articles were assessed for eligibility (phase 2), and 32 were excluded for not meeting the inclusion criteria. Ultimately, 18 studies^3,10^ were included in this systematic review. Details of the search strategy and study selection process are presented in Figure 1. Study selection was performed independently by two reviewers in phases 1 and 2, and disagreements were resolved by discussion and consensus.

**Figure 1:**
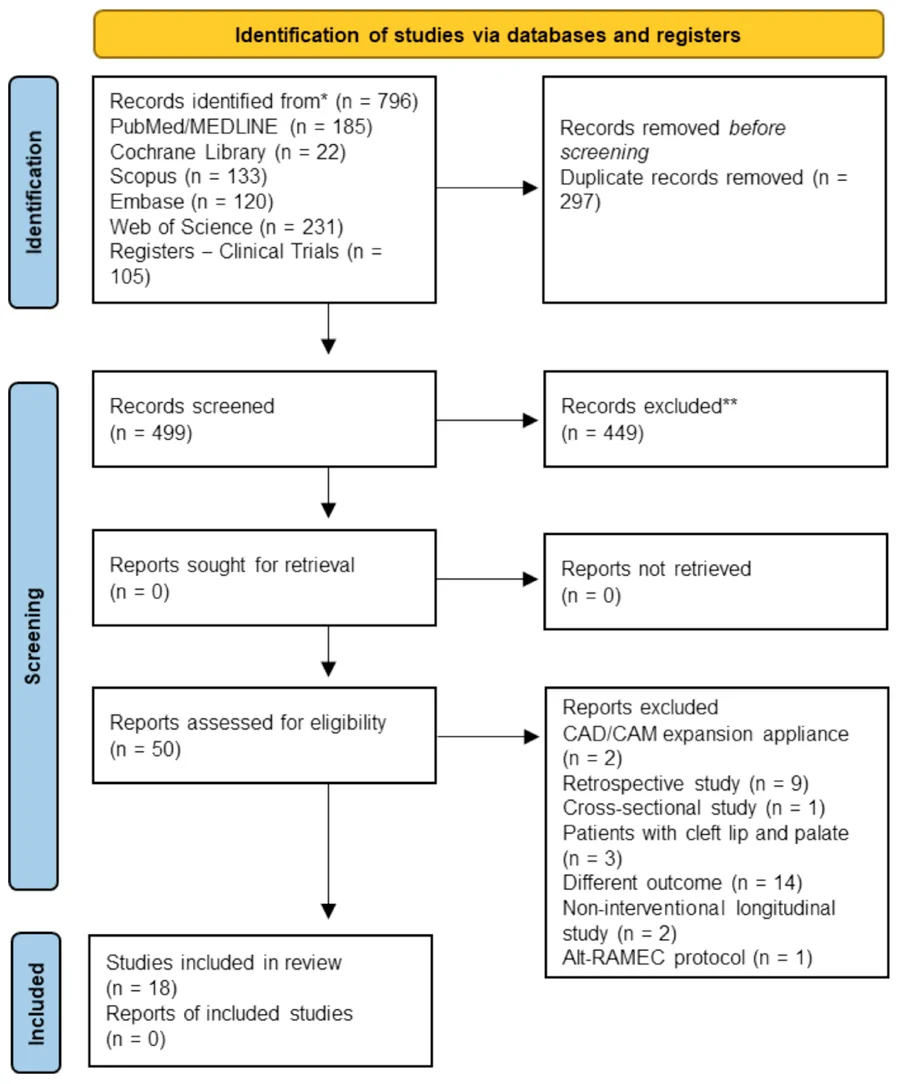
Flowchart detailing the search strategy.

### Study characteristics and results of individual studies

Eighteen studies were included in the qualitative analysis, comprising seven randomized and eleven non-randomized clinical trials (Table 2). The included studies evaluated linear and angular facial soft tissue measurements in a total of 565 children who underwent RME, with mean ages ranging from approximately 8 to 16 years (overall mean age: 11.35 ± 2.2 years). The follow-up period ranged from immediately after active expansion to 18 months after treatment, with most studies reporting outcomes after approximately 6 months of retention. Detailed information on the appliance design and activation protocols is presented in Table 2.

**Table 2:** Characteristics of the included studies.

Author / Year	Type of Study	No. of Patients	Follow-up period	Average Age / Sex	Atresia diagnosis method	Orthodontic appliance	Activation protocol	Facial analysis method	Results	Overall effect
Merlo et al., 2024^10^	RCT	14 patients (RME group)	Immediately before application of the expander (T0) and after one year of retention, immediately after the expander removal (T1).	8.2 ± 1.3 / 6 females and 8 males	Posterior transverse interarch discrepancy: difference between maxillary and mandibular intermolar widths.	Butterfly-shaped stainless-steel	1/4 turn per day (one activation corresponded to 0.2 mm per turn), immediately after the appliance was cemented in place.	3D facial surface images / Face Shape Maxi 3D Scanner Polishape 3D srl	A significant increase was observed in the nasal width (1.3 mm greater compared to the SME group, P = 0.005) and intercanthal width (0.7 mm greater, P = 0.044). Regarding three-dimensional displacements, the nasal marker showed a significant forward movement in the anteroposterior direction (Y-axis) in the RME group, with a mean displacement 0.5 mm greater than the SME group (P = 0.040). The lower lip marker exhibited a significant downward displacement in the vertical direction (Z-axis) in the RME group, with an average difference of 0.9 mm (P = 0.027).	Positive
Dindaroğlu et al., 2016^11^	Non-randomized clinical trials	25 patients (Study group)	Immediately before the appliance was cemented (T0) and just after expansion had been completed (T1).	9.8 / 11 females and 14 males	Unilateral or bilateral posterior crossbite malocclusion requiring RME	Bonded-type expansion appliance	A quarter turn in the morning and evening (0.4 mm/day) until the in the morning and evening (0.4 mm/day) until the palatal cusp of the maxillary first molar came into contact palatal cusp of the maxillary first molar came into contact with the buccal cusp of the mandibular first molar.	3D stereophotogrammetry Image 3dmd / Flex System	For the total face, the mean absolute deviation was 0.54 mm. In the upper face region, the mean deviation was 0.42 mm, with maximum variations close to 1.96 mm. The lower face showed a higher mean absolute deviation of 0.69 mm, with maximum deviations ranging from 1.64 mm to -1.82 mm. The upper lip exhibited a mean deviation of 0.62 mm, with maximum positive and negative deviations of 1.52 mm and -0.95 mm, respectively. The lower lip had a mean deviation of 0.60 mm, with maximum variations of 1.22 mm and -1.10 mm. The nose region showed the least variation, with a mean absolute deviation of 0.41 mm and a maximum negative deviation of -1.51 mm.	Positive
Altındiş et al., 2016^12^	RCT	42 patients (14 Banded RME; 14 Bonded and 14 Modified bonded RME)	Immediately before the appliance was cemented (T0) and just after expansion had been completed (T1).	11.6 ± 13.3 / 24 Female and 18 Male	Class I or II malocclusions and associated moderate maxillary constriction that could be treated with approximately 5 mm of expansion	Banded RME appliance; Bonded RME and Modified bonded RME	One turn of the screw (0.20 mm per turn), and the next day they were placed by the patient’s parent.	3D stereophotogrammetry Image / 3dmd system	After RME, all groups showed a significant increase in the nasal width (Alr–All) and mouth width (Chr–Chl). The band group exhibited a significant increase in upper facial height (Gla–Sn) and changes in the upper lip angle (angular measurement). Both the band and bonding groups showed an increase in the lower lip angle (angular measurement), while a significant increase in lower lip vermilion height (linear measurement) was observed only in the modified bonding group. No significant differences were found between groups in the linear and angular measurements. However, the modified bonding group demonstrated significant volumetric changes in the upper lip (3D volumetric measurement).	Positive
Fastuca et al., 2016^13^	RCT	17 patients (Treated group)	T1, at the beginning of treatment or observation period, and at T2, 18 months apart.	9.8 ± 1.2 / 7 Female and 10 Male 13.4 ± 1.2	Maxillary transverse deficiency with/without cross-bite due to dental compensations and with lack of space for upper central and lateral incisors;	Haas-type expander	The screw of the palatal expander was initially turned twice (0.45 mm initial transversal activation). Afterwards, parents of the patients were instructed to turn the screw once daily thereafter (0.225 mm activation per day).	Facial scanner einscan-Pro / 3D facial scan	After RME, the treated group showed a significant increase in nasal width (Alr–All) and total nasal volume, especially in the nasal dorsum (3D volumetric measurements of total nasal volume and nasal dorsum volume).	Positive
Baysal et al., 2016^14^	RCT	17 patients (Treatment group)	Before treatment (T0) and after 6 months, immediately after the retention/observation period (T1).		*NR	Bonded acrylic splint expander	A quarter turn twice per day (0.5 mm) for the first week, then a quarter turn per day (0.25 mm), until the palatal cusps of the maxillary molar contacted the buccal cusps of the mandibular molar.	3D stereophotogrammetry Image / 3dmd system	After rapid maxillary expansion, no statistically significant differences were found in most soft tissue landmarks, except for the pogonion (pog) point, which showed a significant posterior displacement (-1.33 mm) in the expansion group.	Negative
Alkhayer et al., 2021^15^	Non-randomized clinical trials	25 patients	Before treatment (T0) and at the end of a six-month retention period after the treatment (T1).	11.6 / 13 Female and 12 Male	Maxillary transverse deficiency, assessed both clinically and radiographically, which is associated with either unilateral or bilateral posterior crossbite patients were recalled weekly during the expansion period (2 to 3 weeks).	Hyrax-type expander	2 turns per day (0.5 mm) and the patients were recalled weekly during the expansion period (2 to 3 weeks).	3D facial image / structured-light 3D handheld scanner Artec evatm	Nasal width (Alar R–Alar L) increased by an average of 1.02 mm, while the nasal base width (AcR–AcL) showed an average increase of 1.21 mm. Mouth width (ChR–ChL) increased by 2.62 mm on average, indicating significant expansion in the perioral region. Additionally, significant angular changes were observed: the nasal tip angle (AlarR–Prn–AlarL) increased by 3.2°, and the upper lip angle (ChR–Ls–ChL) and lower lip angle increased by 3.47° and 3.78°, respectively. Regarding soft tissue landmarks, the greatest changes were seen at the right alar point (Alarr: 0.72 mm) and left alar point (Alarl: 0.46 mm), at the subnasale (Sn) with an average displacement of 0.66 mm, and at the right (Chr: 0.46 mm) and left (Chl: 0.66 mm) cheilion points. The pronasale point (Prn) also showed an average displacement of 0.44 mm.	Positive
Altorkat et al., 2016^16^	Non-randomized clinical trials	14 patients	Pre-RME activation (T0) and immediately post-RME expansion (T1).	12.6 ± 1.8 / 7 Females and 7 Males	Either for unilateral or bilateral posterior crossbite correction and/or relief of crowding	Cast cap fixed split acrylic design RME appliance with a Hyrax screw	A quarter turn (0.25 mm) twice a day. The patients were reviewed regularly and expansion was stopped when the palatal cusp of the upper molars was touching the buccal cusp of the lower molars.	3D stereophotogrammetry Image / Di3D system	There was a significant increase in nasal base width (AcR–AcL) by 1.6 mm and in columella width (SnR–SnL) by 0.5 mm. The horizontal displacement angle of the nasal tip (AcR–Prn–AcL) also increased significantly by 3.4°. The nasolabial angle (Prn–Sn–Ls) decreased, but the change was not statistically significant.	Positive
Uzunçıbuk et al., 2023^17^	Non-randomized clinical trials	30 patients (RME experimental group)	The time interval between T1 and T2, representing the initial and intermediate phases, respectively, was approximately 6.3 months	*NR / 16 Female and 14 Male	Presence of unilateral or bilateral posterior crossbite	Hyrax expansion screw	2–3 weeks twice daily (0.25 mm per turn), aiming for a total expansion of 9–10 mm in accordance with the patient’s needs, with a 20% overcorrection.	Frontal and profile photographs / Nikon d7200 camera Equipped with a Sigma 105 macro lens	In the post-expansion group, significant increases were observed in nose width (including Nose Width and Nasal Width of the Middle Third), intercommissural distance, dorsum length of the nose, nasolabial angle, and soft tissue facial angle. There were reductions in alar base width, lower face width, intercanthal distance, and lower lip vermillion length. Upper lip length decreased, particularly in females, while nose length increased more prominently in males.	Positive
dos Santos et al., 2012^18^	Non-randomized clinical trials	20 patients	T1 (pre-expansion), T2 (21 days after T1, at the end of the active expansion phase), and T3 (6 months after T2, totaling approximately 6 months and 21 days after T1).	9.3 / 10 Female and 10 Male	Presence of a narrow maxilla and skeletal posterior crossbite, with indications for RME	Bonded rapid palatal expander	One week after insertion, the expander was activated every 12 hours, corresponding to 0.50 mm/day (two turns/day; 0.25 mm per turn) for 3–4 weeks.	Lateral cephalometric radiographs	The measurements of S line–Ls (position of the upper lip relative to the S line) showed significant protrusion from T1 to T2, followed by retraction from T2 to T3, thus returning to the original position. The S line–Li measurement (position of the lower lip relative to the S line) showed retraction from T1 to T2 and protrusion from T2 to T3. The H line–Prn measurement (distance from the Holdaway line to the pronasale) significantly decreased from T1 to T2. The lower anterior facial height, measured by ANS′–Me′, increased significantly from T1 to T2 and decreased from T2 to T3.	Negative
Akan et al., 2021^19^	RCT	16 patients (RME appliance hyrax)	Before treatment (T0) and immediately after the retention/observation period (T1).	13.4 ± 1.3/7 Female 7 and 9 Male	Bilateral posterior crossbite	Hyrax expansion appliance	Twice a day (2 × 0.25 mm) for the first week and then once per day (0.25 mm). Mean numbers of activations were 25.25 ± 4.42 turns in the Hyrax group.	3D stereophotogrammetry Image / 3dmd system	Following rapid maxillary expansion with the Hyrax appliance (Hyrax RME), significant changes were observed in the facial soft tissues, including an increase in nasal width, mid-face width, the ratio between upper lip vermillion length and lower lip vermillion length, and the upper lip angle.	Positive
	Non-randomized clinical trials	18 patients	Before RME (T1), after maxillary expansion (mean = 0.82 month) (T2), and after retention (mean = 5.95 months)	13.50 ± 1.07 / 15 Female and 3 Male	Maxillary width deficiency and a posterior crossbite	Rigid acrylic bonded appliances	The screw was activated twice daily: one quarter turn in the morning and the other in the evening. The widening procedure was continued until the crossbites were eliminated and 3 mm over-expansion was achieved.	Lateral Cephalometric radiographs	Following rapid maxillary expansion (RME), a significant decrease was observed in the soft tissue facial angle (from 90.63° to 89.48°, p < 0.05), along with significant increases in the H angle (from 7.53° to 9.27°, p < 0.001) and skeletal profile convexity (from 1.64 mm to 3.24 mm, p < 0.001) at T2. During the T3 period, a slight recovery was noted, with a non-significant increase in the soft tissue facial angle and a significant decrease in skeletal profile convexity (p < 0.001), as well as a reduction in the H angle, although not statistically significant.	Positive
Johnson et al., 2010^21^	Non-randomized clinical trials	79 patients	T1, prior to placement of the RME; T2, after completion of active expansion; and T3, after removal of the expander.	13.5 / *NR	*NR	Hyrax expander with bands	One-turn-per-day for an average of 35 days. Each turn of the expansion screw equaled 0.20 mm, which yielded na average expansion of 7 mm.	Digital caliper accurate to 0.01 mm	RME has no significant clinical effects on the widths of the apical base and the greater alar cartilage of the nose; no differences were observed between the two maturational subgroups.	Negative
Berger et al., 1999^3^	Non-randomized clinical trials	20 patients	Initial, bond appliance, stop expansion, debond appliance, and 1 year retention.	8.6 / 9 Female and 11 Male	Unilateral and bilateral posterior crossbites were treated	Bonded acrylic hyrax	Once per day in both groups. The average amount of expansion was 4.0 mm in the orthopedic group.	Frontal photographs / 90 mm macro lens	The measurements of upper lip length, lower lip-chin length, upper face width, lower face width, upper lip vermilion, and lower lip vermilion demonstrated changes during treatment but showed no significant change from initial values after 1 year of retention. Overall face height, intercanthal distance, average eye width, and nose length did not change over time. Soft tissue nasal width increased 2.0 mm during treatment.	Negative
Ozturk et al., 2022^22^	Non-randomized clinical trials	20 patients	Pretreatment (T0), Postdistraction (T1), and Postconsolidation 165.85 ± 10.42 days (T2)	15.86 ± 2.17	Narrow and V-shaped mandibular dental arch, moderate (4–6 mm) or severe (6–10 mm) mandibular anterior dental crowding, a transverse deficiency in the lower and upper jaw, dark buccal corridors during smiling, and single- or double-sided lingual crossbite closure in the lower jaw	Hyrax screw	Activated once a day (0.2 mm). The activation period lasted 9.19 ± 0.81 days, and the activation amount was an average of 8.12 ± 1.78 mm.	3D stereophotogrammetry Image / 3dmd system	Nasal and mouth widthincreased after RME as compared with baseline (P <.05). Upper and lower lip angles increased after RME (P < .05).	Positive
Iwasaki et al., 2021^23^	RCT	21 patients (Banded RME)	Initial (T0) and final (T1, 6 months at appliance removal)	13.7 / 11 Female and 10 Male	Distance from the palatal cusp of the first permanent maxillary molars being more than 5mm less than the distance of the central grooves of the mandibular maxillary molars being more than 5mm less than the distance of the central grooves of the mandibular first permanent molars measured clinically	Hyrax	Activation protocol was 2 turns/day (total 0.5 mm) with weekly follow-up appointments until posterior cross-bite overcorrection (palatal cusps of the maxillary first molars being in contact with the buccal cusps of the mandibular first molars) to account for post-expansion relapse.	3D images to perform Measurements / medical image analyzing system (ImagnosisVE, Imagnosis	The Hyrax group showed significant increases in anterior (10.7%) and posterior (8.2%) nasal width, indicating moderate but statistically significant changes in facial soft tissues.	Positive
Santariello et al., 2014^24^	Non-randomized clinical trials	61 patients (RME)	T0 – prior to the placement of RME; T1 – after completion of active expansion phase; T2 – at the removal of the expander (nearly 6 months after T0).	10.5 ± 1.8 years / 26 Female and 35 Male	*NR	Hyrax type expander	*NR	Caliper	In the RME study group, there was a statistically significant increase in the width of the greater alar cartilage (GAC) (0.8 ± 0.2 mm, P < 0.0001).	Positive
Badreddine et al., 2022^25^	Non-randomized clinical trials	104 patients	(T1) pre-RME and (T2) after expander removal	10.1 ± 2.10 years / 62 Male and 42 Female	*NR	Hyrax expander	6/4 initial activation and 2/4 daily activations until compatibility of the upper alveolar bone base with the lower WALA edge (area of greater transverse width of the mandibular arch in its buccal face)	Multislice computed tomography using the Dolphin Imaging 11.7 Premium software	The results of the global analysis showed that, in the EG, there were significant increases (p < 0.05) in all variables evaluated between moments T1 and T2, in which the Alar Width (AW) and the Soft Tissue Insertion Width (STIW) presented relevant increases of 7.0% and 6.5% respectively. Changes in Volume (VNT) and Area (ArT) also showed considerable increases of 36.3% and 27.5%, respectively.	Positive
Poiani et al., 2026^26^	RCT	22 patients (11 EDO; 11 HT)	Pre-expansion (T1), immediately post-expansion (T2), and 6 months post-expansion (T3)	EDO 9.45 years / 8 Females and 3 Males; HT 9.35 years / 7 Females and 4 Males	Class I or II malocclusions and unilateral or bilateral posterior crossbites	Expander with Differential Opening (EDO) and the Hyrax-type Expander (HT)	In the EDO group both screws were activated for 9 days following a protocol of two quarter turns (0.4 mm) in the morning and two quarter turns in the evening. The anterior screw was further activated for an additional 4 days, following the same protocol. The total activation was 7.2 mm for the posterior screw and 10.4 mm for the anterior screw. In the HT group the single screw was activated for 9 days, following the same activation protocol, reaching a 7.2 mm activation	The LifeViz™ Mini system uses stereophotogrammetric technology	Both expanders significantly increased nasal base width immediately after expansion (EDO: +2.68 mm; HT: +1.33 mm; P < 0.001), with no significant difference between groups. During the retention period, a significant partial relapse was observed only in the EDO group (−1.14 mm), while the HT group remained stable. Intercanthal width showed a small but significant increase only in the EDO group (+0.62 mm), whereas no significant changes were observed in the HT group (P = 0.477). Labial intercommissural width increased significantly in both groups (EDO: +2.26 mm; HT: +2.49 mm; P < 0.05), with stability during retention. Both upper and lower facial heights increased slightly but significantly after expansion, with no differences between groups and stability over time (P > 0.05).	Positive

ME: rapid maxillary expansion, RCT: randomized clinical trial, SME: slow maxillary expansion, 3D: three-dimensional. ^a^Lack of control for confounding factors and insufficient reporting of blinding, participant selection, and missing data in some non-randomized studies may have introduced bias. ^b^Heterogeneity among the variables used to assess the effects of RME on facial soft tissues, with widely differing linear and angular measurements, may have contributed to the divergent results observed across studies. ^c^Total sample sizes below recommended thresholds (RCTs: 147; non-randomized: 312) and heterogeneity in linear and angular soft tissue measurements increase uncertainty of effect estimates.

A diagnosis of transverse maxillary deficiency was explicitly reported in only two studies.^10,23^ One study defined the condition based on posterior transverse interarch discrepancy, calculated as the difference between maxillary and mandibular intermolar widths,^10^ whereas another defined it clinically as a maxillary intermolar distance more than 5 mm smaller than the distance between the central grooves of the mandibular first permanent molars.^23^ Four studies did not report the specific diagnostic criteria used to establish this diagnosis.^14,21,24,25^ In the remaining studies, the diagnosis was primarily based on the occlusal characteristics or clinical measurements, including unilateral or bilateral posterior crossbite,^3,11,15,26^ maxillary transverse constriction associated with crowding or clinical indications for RME,^12,16,18,19^ skeletal posterior crossbite associated with a narrow maxilla,^17,20^ and transverse discrepancies identified through clinical measurements such as posterior interarch discrepancy or intermolar width differences.^13,22^

#### Effects according to appliance design

The 18 included studies employed three main categories of expanders: tooth-borne expanders (e.g., Hyrax-type expander, Hyrax screw, banded RME appliance, butterfly-shaped stainless-steel expander and Expander with Differential Opening (EDO)),^10,12,15,17,19,22-26^tooth-borne expanders with acrylic coverage (e.g., bonded RME appliance, modified bonded RME, bonded acrylic splint expander, bonded rapid palatal expander, rigid acrylic bonded appliance, and bonded acrylic Hyrax),^3,11,12,14,16,18,20^ and tooth–tissue-borne expanders (e.g., Haas-type expander).^13^

Studies using tooth-borne (banded) appliances consistently reported an increase in the nasal and perioral dimensions following RME, with mean expansions ranging from 1.0 to 1.3 mm in nasal width and approximately 2.5 mm in mouth width.^10,12,15,17,19,22,23,24^ Mild angular changes (±3–4°) were observed in the lips and nose, along with small displacements of facial soft tissue landmarks. Significant increases were reported in nasal width (Alr–All), mouth width (Chr–Chl), upper and lower lip angles, and upper facial height (Gla–Sn), as well as volumetric changes in the upper lip in some groups, with additional evidence of soft tissue expansion and volumetric and surface area increase in the nasolabial region.^12,25^ Additional findings included increase in the nasal width (including the middle third), intercommissural distance, dorsum length of the nose, nasolabial angle, and soft tissue facial angle, along with reductions in the alar base width, lower facial width, intercanthal distance, lower lip vermillion length, and upper lip length.^17^More recently, significant increase in the nasal base width (≈1.3 mm) and intercommissural width (≈2.5 mm) have also been reported, along with slight increase in the upper and lower facial heights; these measurements remained stable during the retention period, with no significant changes in the intercanthal width for Hyrax-type appliances.^26^

In contrast, studies using bonded expanders demonstrated milder and predominantly transient facial soft tissue changes, particularly in the nasal and labial regions, with small 3D variations (±0.4–0.7 mm) and a slight increase in the nasal and mouth width.^11,12,16^ Temporary changes in lip position relative to the S line (S-line–Ls and S-line–Li), a slight increase in facial convexity, and an increase in the H angle were observed after expansion, with a tendency toward partial regression during the post-expansion follow-up period.^18,20^Overall, no clinically significant or permanent effects were identified, suggesting that bonded appliances produce subtle and reversible facial soft tissue modifications.^3,11,12,14,16,18,20^

Finally, the Haas appliance was associated with a significant increase in the nasal width and total nasal volume after RME, particularly in the nasal dorsum region, as demonstrated by 3D volumetric measurements.^13^

#### Effects according to activation protocol and magnitude of expansion

With respect to the activation protocol, only a limited number of studies reported the total amount of expansion achieved.^3,19,21,22,26^ Among these, Uzunçıbuk et al.^17^ described two daily activations of 0.25 mm for 2–3 weeks, resulting in a total expansion of 9–10 mm with 20% overcorrection. Akan et al.^19^ performed two daily activations (2 × 0.25 mm) during the first week, followed by one daily activation (0.25 mm), with a mean of 25.25 ± 4.42 turns performed in the Hyrax group. Johnson et al.^21^ applied one activation per day for approximately 35 days, corresponding to a mean expansion of 7 mm. Berger et al.^3^ reported daily activations with a mean expansion of 4.0 mm in the orthopedic group, whereas Ozturk et al.^22^ reported a mean expansion of 8.12 ± 1.78 mm. Similarly, Poiani et al.^26^ reported a total activation of 7.2 mm posteriorly and 10.4 mm anteriorly in the EDO group, whereas the Hyrax group achieved a total activation of 7.2 mm following a comparable activation protocol.

Despite this variability (approximately 4 to 10 mm of expansion), no clear association could be established between the magnitude of expansion and the extent of facial soft tissue changes. Even in studies reporting larger expansions, the observed soft tissue modifications remained small in magnitude.

Conversely, the remaining studies^10-18,20,23,25^did not report the total amount of expansion achieved; however, they described clinical criteria for terminating activation. These criteria included cusp-to-cusp contact, defined as the point at which the palatal cusps of the maxillary molars contacted the buccal cusps of the mandibular molars, or the correction of posterior crossbite. One study^24^ did not report either the magnitude of expansion or the criteria used to discontinue activation.

#### Methods of facial analysis

Regarding facial analysis methods, six studies^11,12,14,16,19,22,26^employed 3D stereophotogrammetry, while three studies^10,13,15^used structured-light 3D scanning. Two studies^3,17^ used frontal and profile photographs, and two studies^18,20^used lateral cephalometric radiographs. Additionally, two studies performed manual measurements using calipers,^21,24^ and two studies^23,25^ used cone-beam computed tomography (CBCT) to generate 3D models analyzed using specialized software.

#### Summary of soft tissue changes

Regarding linear measurements, a consistent increase in nasal and perioral dimensions was observed. Nasal width increased by approximately 1.0–1.6 mm in most studies;^10,12,15 4^ the increase was 1.3 mm in the study by Baysal et al. (p = 0.005) and up to 2.0 mm in some bonded-appliance protocols.^3^ Mouth width increased by approximately 2.5–2.6 mm,^12,15,17,19,22^while nasal base width increased by approximately 1.2–1.6 mm. A significant increase in the greater alar cartilage width was also reported (0.8 ± 0.2 mm; p < 0.0001).

Alar width and soft tissue insertion width also increased by 7.0% and 6.5%, respectively (p < 0.05),^25^ while volume and surface area of the nasolabial region increased by 36.3% and 27.5%, respectively (p < 0.05). Immediate nasal base widening after expansion was also reported (EDO: +2.68 mm; HT: +1.33 mm; p < 0.001), and intercommissural width increased significantly in both groups (EDO: +2.26 mm; HT: +2.49 mm; p < 0.05).^26^

Additional changes included a slight increase in upper facial height and nasal length,^12,17^ as well as a localized reduction in the alar base width and lower facial width following the active expansion phase.^17^ 3D studies demonstrated small displacements of the subnasale (±0.6 mm), pronasale (±0.4 mm), and labial commissures (±0.5–0.7 mm),^15^ reinforcing the limited magnitude of the observed morphological changes.

With respect to angular measurements, the results demonstrated subtle variations, with mean increases of approximately ±3–4° in the upper and lower lip angles (p < 0.05),^15,19,22^ as well as widening of the nasal angle (±3°) and the nasolabial angle, suggesting mild projection of the nasal tip.^15,17,19^A temporary decrease in the soft tissue facial angle (p < 0.05) and an increase in the H angle (p < 0.001) were also observed, indicating a transient increase in facial convexity with a tendency toward regression following the retention phase.^18,20^ In groups treated with bonded appliances, lip protrusion followed by repositioning relative to esthetic reference lines (S line and H line) was noted, further supporting the reversibility of these soft tissue changes.^18,20^

## Risk of bias within the studies

According to the RoB 2.0 tool, most of the included randomized clinical trials were judged to have a low risk of bias,^10,13,14,19,23,26^ as they reported appropriate randomization procedures, clearly defined eligibility criteria, and standardized outcome assessments. However, the study by Altındiş et al.^12^ was classified as having some concerns, mainly due to insufficient reporting regarding methodological aspects. Specifically, the study did not report details about blinding procedures for outcome assessment, which raised concerns in Domain 4 (bias in measurement of the outcome). In addition, the absence of information regarding trial registration, CONSORT reporting, and participant losses limited the assessment of transparency and completeness of reporting (Figure 2).

**Figure 2:**
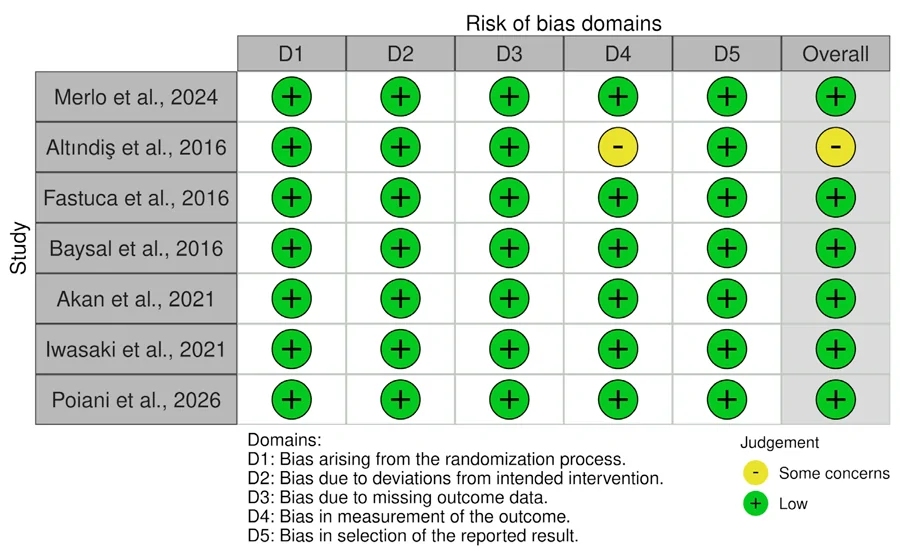
Risk of bias assessment of the included randomized studies using the RoB 2 tool.

Among the non-randomized prospective studies evaluated using ROBINS-I, five studies were judged to have a moderate risk of bias,^3,11,17,18,20,24^ whereas five were classified as having a serious risk of bias.^15,16,21,22,25^ The main reason for the higher risk-of-bias judgements was the potential for confounding inherent to non-randomized before-and-after study designs, particularly in studies without adequate control for growth- and time-related factors. Additional concerns included limitations in participant selection, incomplete reporting of methodological procedures, insufficient information regarding intervention classification, and missing information on participant flow and outcome assessment. No non-randomized study was judged to have an overall low risk of bias (Figure 3).

**Figure 3:**
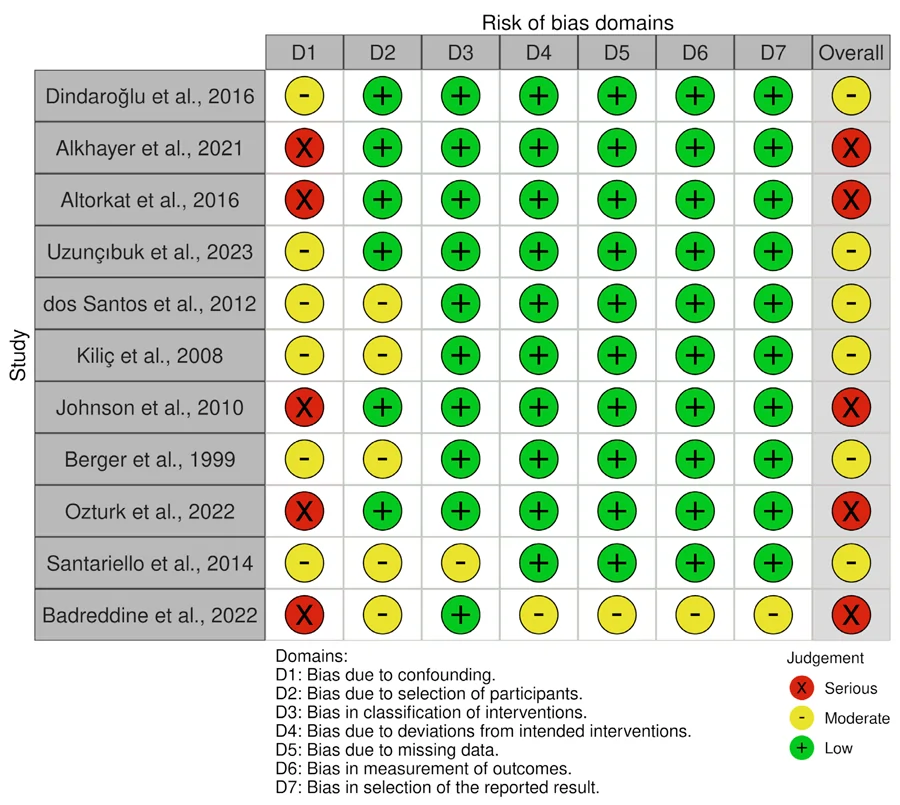
Risk of bias assessment of the included non-randomized studies using the ROBINS-I tool.

## Quantitative synthesis

Due to heterogeneity in the study designs, intervention protocols and outcome measures, a formal quantitative synthesis was not conducted.

## Certainty of evidence

The GRADE assessment classified the certainty of the evidence as low and very low for randomized and non-randomized clinical trials, respectively. Downgrading was applied in the domains of inconsistency, which was rated as serious for both study types owing to heterogeneity in the outcomes arising from the use of multiple assessment methods, and imprecision, which was rated as serious due to relatively small sample sizes and variability in the measurements of facial soft tissue changes. Additionally, for non-randomized studies, the risk of bias was rated as serious owing to limitations in the study design, including lack of control for confounding factors and insufficient reporting of methodological aspects such as blinding and missing data (Table 3).

**Table 3:** Certainty of the evidence assessment – facial soft tissue changes.

No of studies	Study design	Risk of Bias	Inconsistency	Indirectness	Imprecision	Other considerations	Important				Certainty
7	RCT	Not serious	Serious^b^	Not serious	Serious^b^	None	Six studies will present a positive effect of the intervention^10^ ^,^ ^12^ ^,^ ^13^ ^,^ ^19^ ^,^ ^23^ ^,^ ^26^				⨁⨁◯◯
											Low
No of studies	Study design	Risk of Bias	Inconsistency	Indirectness	Imprecision	Large effect	Dose response	Residual confounders	Other considerations	Important	Certainty
11	Non-randomized clinical trials	Serious	Serious^b^	Not serious	Serious^c^	Not serious	Not serious	Not serious	None	Eight studies will present a positive effect of the intervention^11^ ^,^ ^15^ ^,^ ^16^ ^,^ ^17^ ^,^ ^20^ ^,^ ^22^ ^,^ ^24^ ^,^ ^25^	⨁◯◯◯
											Very Low

^a^ Lack of control for confounding factors and insufficient reporting of blinding, participant selection, and missing data in some non-randomized studies may have introduced bias; bHeterogeneity among the variables used to assess the effects of RME on facial soft tissues, with widely differing linear and angular measurements, may have contributed to the divergent results observed across studies; cTotal sample sizes below recommended thresholds and heterogeneity in linear and angular soft tissue measurements increase uncertainty of effect estimates.

## Discussion

This systematic review evaluated whether appliance design and the magnitude of RME influence facial soft tissue changes in children with maxillary constriction. The topics related to the impact of RME on facial soft tissues and the influence of expansion magnitude are discussed below. In addition, the main methods used for facial assessment are detailed, highlighting their relevance to result accuracy, appropriate technique selection, and interpretation of the observed changes, as well as the limitations and future perspectives for further research.

### Impact of RME on Facial Soft Tissues

Most included studies reported measurable facial soft tissue changes following RME, demonstrating that RME reported a significant increase in nasal base width.^10,12,13,15-17,22-24,26^ In addition, discrete changes were observed in the soft tissues of both the upper and lower face. Some authors reported small variations in facial morphology, generally less than 1 mm in the facial and labial regions,^11^ accompanied by an increase in the nasal width, changes in the midfacial region, and modifications in the proportions and angles of the upper and lower lips.^19,22^A similar increase in the nasal width and soft tissue dimensions have also been reported.^25^

Facial morphological changes were also described after the retention period, including remodeling of the facial profile,^15,20^ suggesting possible modifications in the H angle and profile convexity, with evidence of stability in nasal and perioral dimensions over time following expansion.^26^ In the nasolabial complex, Altorkat et al.^16^ reported an increase in the nasal base length associated with nasal retraction and flattening, with individual variability among patients. Similarly, Öztürk et al.^22^ identified an increase in the mouth length and in two angular measurements of the upper and lower lips, as well as a reduction in the distance from the lower lip to the E plane, a parameter used to assess labial projection in the facial profile.

Conversely, some studies did not identify significant changes in the facial soft tissues following RME.^3,14,18,21^ Despite the absence of observable changes, the randomized clinical trial conducted by Baysal et al.^14^ exhibited a low risk of bias, supporting the reliability of its findings. In contrast, although Johnson et al.^21^ also reported no significant facial soft tissue changes, this non-randomized study was judged to have a serious risk of bias, mainly owing to concerns regarding confounding inherent to its study design. Therefore, its findings should be interpreted with caution. Nevertheless, the consistency of these findings across studies suggests that in certain clinical contexts, RME may not produce clinically relevant alterations in facial soft tissues.

Additional studies, such as that by dos Santos et al.,^18^ also reported no significant modifications in the facial profile, including apical base length and upper nasal cartilage length. However, these findings should be interpreted with caution, as the study was judged to have a moderate risk of bias according to the ROBINS-I assessment, primarily due to concerns regarding confounding and participant selection, which are inherent limitations of non-randomized study designs.

Similarly, Berger et al.^3^ observed stability in total facial height, intercanthal distance, mid-eye length, and nasal compression over time. Although an increase of approximately 2.0 mm in the nasal width was observed, the clinical significance of this change remains uncertain, as small statistically significant differences may not necessarily translate into perceptible changes in facial appearance. Nevertheless, this study was judged to have a moderate risk of bias according to the ROBINS-I assessment, mainly owing to concerns regarding confounding and participant selection, reinforcing the need for cautious interpretation. Nonetheless, these findings corroborate the weak correspondence between skeletal changes induced by RME and the response of facial soft tissues, which demonstrate high adaptive capacity and tend to attenuate underlying structural modifications.

### Influence of appliance design and expansion magnitude

Although studies suggest that different types of expanders produce distinct patterns of force transmission, the changes observed in facial soft tissues were generally subtle. Banded appliances, such as the Hyrax, tend to promote slightly more consistent changes in the nasal width and perioral region,^10,12,15,17,19,22-24^ whereas bonded expanders distribute forces more homogeneously across the palate, resulting in more subtle labial modifications.^3,11,12,14,16,18,20^ Haas-type appliances, which are tooth–tissue-borne, may produce additional volumetric effects in the upper lip and alar regions; however, this response shows considerable interindividual variability.^13^

Despite these biomechanical differences, no study identified clinically relevant changes attributable exclusively to expander design. Even when different appliances were compared, the observed differences disappeared when similar expansion magnitudes were achieved, indicating that other factors, such as total expansion amount, activation protocol, and individual growth characteristics, exert a more decisive influence on facial soft tissue changes. This is further supported by evidence comparing the EDO and Hyrax-type appliances, in which a similar increase in the nasal and perioral dimensions was observed despite differences in expansion mechanics.^26^ Thus, while appliance design modulates force distribution and the magnitude of measurable changes, it does not appear to be a critical determinant of esthetically significant effects.

In this context, expansion magnitude emerges as a potentially more relevant variable. Moderate activation protocols, such as the 6-mm expansion evaluated by Fastuca et al.,^13^ did not demonstrate a correlation between skeletal changes and soft tissue modifications, whereas larger expansions of up to 14 mm^14,15^ also failed to produce clinically perceptible effects. These findings indicate that although skeletal expansion is essential for modifying the osseous base, facial soft tissues exhibit elastic behavior and adaptive capacity that limit the transfer of these changes to the facial profile.

Nevertheless, detailed analyses suggest a possible dose–response relationship, whereby greater or more frequent activations result in more detectable changes. Merlo et al.^10^ reported an increase in the nasal width and anterior displacement of soft tissue landmarks with activations of 0.2 mm/day, whereas Alkhayer et al.^15^ reported more pronounced increases in the nasal width, nasal base width, and mouth width with activations of 0.5 mm/day for 2–3 weeks. In contrast, studies employing smaller or slower activations, such as those by Baysal et al.^14^ and Johnson et al.,^21^ reported minimal changes. This pattern is further supported by Poiani et al.,^26^ who demonstrated that higher total activation magnitudes (up to 10.4 mm anteriorly in the differential expander group) were associated with measurable soft tissue changes, whereas lower activation magnitudes (7.2 mm in the Hyrax group) contributed to more limited effects.

Importantly, these changes tend to be transient even when present. Altorkat et al.^16^ demonstrated that modifications in lip position, facial height, and vermilion volume were not maintained after 1 year, reinforcing the adaptive nature of facial soft tissues during ongoing growth. The GRADE assessment indicated low and very low certainty of evidence for randomized and non-randomized clinical trials, respectively, which limits confidence in these findings. Despite this, the functional and transverse benefits of RME are considered to clearly outweigh any potential esthetic impact. Most of the included studies assessed soft tissue changes at the end of the consolidation or retention period, generally around 6 months post-expansion, which was the most commonly reported follow-up time point. While some studies included longer-term follow-ups (up to 12–18 months), the majority of reported changes were observed during this 6-month period. Therefore, the term “transient” reflects refers to changes that were small and tend to stabilize over time rather than changes that necessarily disappeared immediately.

Overall, the findings indicate that while appliance design influences the force distribution patterns and magnitude of measurable changes, the intensity and frequency of expansion appear to exert a greater influence on facial soft tissue alterations, which remain small, transient, and rarely perceptible. Consequently, control of the activation protocol plays a more important role in the predictability of facial effects than the type of expander used.

Nevertheless, an important gap remains in the literature, as no study to date has evaluated whether facial soft tissue changes resulting from RME are actually perceived as relevant by patients or by lay observers. Although all studies included in this review quantified linear and angular changes,^3,10^ whether these minimal soft tissue modifications have a real impact on esthetic perception or on the way faces are visually processed remains unclear.

Thus, evidence from eye-tracking studies indicates that human visual attention is not randomly distributed; rather, it tends to focus on regions of greater visual salience, particularly in the presence of facial asymmetries or dentomaxillofacial deformities.^27^ Incorporating this approach could help clarify whether the subtle changes associated with RME, such as variations in the nasal width, lip projection, or perioral regions, actually capture observer attention or remain visually imperceptible, as suggested by current findings.

### Diagnostic tools for the assessment of facial soft tissues

The analysis of the assessment methods used in the included studies shows that 3D stereophotogrammetry was the most commonly applied technique, particularly using the 3dMD system,^11,12,14,19,22^ with several studies reporting positive effects on facial soft tissues.^11,16,19,22^ However, Altındiş et al.^12^ and Baysal et al.,^14^ despite employing the same method, did not observe clinically relevant changes. Although stereophotogrammetry offers high accuracy and sensitivity, it captures only the external facial geometry and does not quantify internal structures or the thickness of soft tissues.^14^ Nevertheless, by surpassing conventional methods such as direct anthropometry and 2D photography, it is widely recommended for facial assessment in reconstructive and orthognathic contexts owing to its superior precision and reproducibility.

In addition, several studies employed structured light scanners, which project light patterns onto the facial surface to reconstruct 3D models, also identifying measurable facial soft tissue changes following RME.^10,13,15^ These findings reinforce the potential of such technologies to detect soft tissue changes induced by RME, even when these changes are not clinically expressive, supporting the high sensitivity of both 3D systems. Structured light scanners have been shown to provide greater accuracy in the midfacial region compared with direct anthropometric measurements, particularly when images are captured from multiple angles in less than 1 second.^15^

Currently, 3D methods provide a more faithful representation of facial surface morphology, allowing the identification of subtle changes that are often not captured by 2D examinations or direct measurements. This higher sensitivity underscores the importance of selecting appropriate imaging techniques for accurately assessing the effects of RME. In this context, only two studies included in this review^23,25^ used CBCT for generating 3D models. The study using CBCT^23^ reported a significant increase in the anterior nasal length in the group treated with a Hyrax expander, highlighting the potential of CBCT-based 3D imaging for the precise detection of structural changes. Complementarily, multislice computed tomography with dedicated 3D reconstruction software (Dolphin Imaging 11.7 Premium)^25^ has also been used for 3D reconstruction of facial structures.

Studies using 2D techniques, such as calipers^21^ and photographs^3^, generally did not detect significant changes in soft tissues, largely due to methodological limitations. However, Santariello et al.^24^ and Uzunçıbuk et al.^17^ reported subtle but measurable changes after RME, suggesting that even 2D methods may identify soft tissue alterations depending on the protocol and parameters analyzed, although these effects may lack clinical relevance.

Studies employing lateral cephalometric radiographs reported divergent findings regarding the effects of RME on facial soft tissues. While dos Santos et al.^18^ found no significant profile changes, Kiliç et al.^20^ reported an increase in facial convexity and the H angle, followed by partial regression after the retention period. Nevertheless, both studies were judged to have a moderate risk of bias, reflecting methodological limitations inherent to non-randomized study designs, which may compromise the reliability of these estimates and further emphasize the limitations of cephalometry in detecting subtle facial soft tissue changes.

### Limitations and future recommendations

This systematic review presents important limitations. Only a limited number of studies were registered and adhered to appropriate methodological guidelines, and substantial heterogeneity was observed among them, both in terms of the types of appliances used and the magnitude of expansion performed. In addition, the included studies employed different parameters to assess linear and angular changes, which precluded outcome standardization and, consequently, data pooling for meta-analysis. Furthermore, the scarcity of studies including control groups limited direct comparisons capable of isolating the true effects of the intervention and limited the ability to perform meaningful comparative analyses between treated and control groups. In addition, the follow-up period reported in most of the included studies was limited to 6 months, making it unclear whether the observed soft tissue changes are transient or remain stable over time. Most of the available evidence originated from non-randomized before-and-after studies, which are inherently susceptible to confounding due to growth and other time-related factors. Consequently, the overall risk of bias of these studies was predominantly judged as moderate or serious, reducing the certainty of the available evidence.

A lack of clearly defined diagnostic criteria for maxillary constriction was also observed, which limits the ability to assess the stability of facial soft tissue changes. The adoption of standardized diagnostic criteria would allow more accurate comparisons and greater validity and comparability of the results, providing a more comprehensive and long-term understanding of treatment effects, particularly considering that the primary rationale for the therapeutic intervention is the correction of transverse maxillary deficiency.

Although 3D analyses predominated, there was a lack of standardization in the measurement methods, including reference landmarks, variables, and assessment tools, which hindered comparisons across studies. The use of standardized imaging protocols, blinded assessments, and validated scales for esthetic and functional impact may help identify clinically meaningful changes. Longitudinal studies combining subjective perception, visual attention patterns, and 3D measurements are essential for determining whether the observed changes have true clinical and esthetic relevance or whether their impact is merely measurable yet imperceptible from the patient’s perspective.

## Conclusion

Within a 6-month follow-up period, banded expanders may cause slightly greater changes in the nasal and perioral regions; however, these effects appear to be primarily related to the greater magnitude of expansion rather than to appliance design itself. When similar amounts of expansion are achieved, these differences tend to disappear, indicating that expansion magnitude may be a more important determinant of facial soft tissue changes than appliance design. Nevertheless, longitudinal studies integrating subjective perception, visual attention patterns, and 3D analyses are essential for determining whether these changes have true clinical and esthetic relevance or whether their impact is merely measurable but imperceptible to patients.

## Data Availability

The datasets generated during and/or analyzed during the current study are available from the corresponding author on reasonable request.
